# Metagenomic insights into the antibiotic resistomes of typical Chinese dairy farm environments

**DOI:** 10.3389/fmicb.2022.990272

**Published:** 2022-09-28

**Authors:** Jijun Kang, Yiming Liu, Xiaojie Chen, Fei Xu, Honglei Wang, Wenguang Xiong, Xiubo Li

**Affiliations:** ^1^Key Laboratory of Animal Antimicrobial Resistance Surveillance, Ministry of Agriculture and Rural Affairs, Feed Research Institute, Chinese Academy of Agricultural Sciences, Beijing, China; ^2^Laboratory of Quality and Safety Risk Assessment for Products on Feed-origin Risk Factor, Ministry of Agriculture and Rural Affairs, Feed Research Institute, Chinese Academy of Agricultural Sciences, Beijing, China; ^3^Guangdong Provincial Key Laboratory of Veterinary Pharmaceutic Development and Safety Evaluation, South China Agricultural University, Guangzhou, China

**Keywords:** antibiotic resistance genes, metagenomic, dairy farm, feces, wastewater, soil, microbiome, bacterial host

## Abstract

Antibiotic resistance genes (ARGs) in the environment pose a threat to human and animal health. Dairy cows are important livestock in China; however, a comprehensive understanding of antibiotic resistance in their production environment has not been well clarified. In this study, we used metagenomic methods to analyze the resistomes, microbiomes, and potential ARG bacterial hosts in typical dairy farm environments (including feces, wastewater, and soil). The ARGs resistant to tetracyclines, MLS, β-lactams, aminoglycoside, and multidrug was dominant in the dairy farm ecosystem. The abundance and diversity of total ARGs in dairy feces and wastewater were significantly higher than in soil (*P* < 0.05). The same environmental samples from different dairy have similar resistomes and microbiomes. A high detection rate of *tet*(X) in wastewater and feces (100% and 71.4%, respectively), high abundance (range from 5.74 to 68.99 copies/Gb), and the finding of *tet*(X5) challenged the clinical application of the last antibiotics resort of tigecycline. Network analysis identified *Bacteroides* as the dominant genus in feces and wastewater, which harbored the greatest abundance of their respective total ARG coverage and shared ARGs. These results improved our understanding of ARG profiles and their bacterial hosts in dairy farm environments and provided a basis for further surveillance.

## Introduction

The emergence of antibiotic resistance genes (ARGs) has raised global concerns. Extensive use of antibiotics during animal production was credited with the rapid spread of antibiotic resistance and posed an increasing threat to public health (Hu et al., 2017; Dyar et al., 2018). It is estimated that the global antibiotic use in food animals will increase by 11.5%, from 93,309 tons in 2017 to 104,079 tons in 2030 (Tiseo et al., 2020). As the largest antimicrobial producer and consumer worldwide, China faces a growing crisis created by ARGs (Qiao et al., 2018).

Vancomycin, colistin, and tigecycline are the last defenses against infection by multidrug-resistant pathogens. However, in recent years, different vancomycin resistance genes, such as *van*(A), *van*(C), *van*(N), and the plasmid-mediated colistin resistance gene *mcr-1*, have been discovered in humans, animals, and their surrounding environments (Al-Tawfiq et al., 2017; Ahmed and Baptiste, 2018). With the increasing challenges of vancomycin and colistin clinical usage, tigecycline has become the most important therapeutic used to treat serious infections caused by extensively resistant bacteria, e.g., vancomycin-resistant *Enterococcus* strains, methicillin-resistant *Staphylococcus* aureus and carbapenem-resistant *Enterobacteriaceae* (Pournaras et al., 2011; Knafl et al., 2016). *Tet*(X), an inactivation enzyme, can trigger resistance to tetracyclines by catalyzing the oxygen-dependent degradation of the drugs (Yang et al., 2004). Some of its variants, such as *tet*(X3), *tet*(X4), and *tet*(X5), can mediate a high level of tigecycline resistance through plasmids and cause a threat to the use of this last-resort antibiotic in both humans and animals(He et al., 2019).

The farm environment is a hotspot of ARGs and an important location for ARGs transfer from animals to humans. ARGs in animal feces can directly enter environments including farmland soil, surface water, and groundwater, and then be conveyed to humans through the food chain or by airborne dissemination (Sui et al., 2016; Zhang et al., 2019). Antibiotic-containing residues in animal wastes could create selection pressure on the microbiome and increase the selection of ARGs (Zhu et al., 2019). ARGs have been reported in different environments, such as manure, soil, wastewater, sediments, and air from livestock, poultry, and aquaculture farms (Noyes et al., 2016; Sancheza et al., 2016). ARGs host bacteria, which harbor mobile genetic elements including plasmids, transposons, and integrons, exert a crucial role in ARG spread *via* horizontal gene transfer (HGT) (Zhu et al., 2019). Bacteria community shifts are often accompanied by changes in ARG profiles (Jia et al., 2015).

On conventional dairy farms, manure and wastewater are often used to fertilize crop fields due to their nutrient richness. Consequently, ARGs included in dairy wastes can diffuse into the soil and enter cultivated plants (Tien et al., 2017). This initiates the diffusion chain from animal to human through the environment. To reduce costs and provide comfort to cows, some farms recycle the solids of manure and wastewater for use as cow bedding after solid–liquid separation, digestion, and composting (Leach et al., 2015). This practice can lead to the circulation of ARGs and their dissemination between animals and the environment. Infections caused by ARG host bacteria, especially by the bacteria containing multidrug resistance genes (e.g., *cfr*), can increase the therapeutic difficulty and create more economic losses to dairy farms. Mastitis is the major disease in the dairy industry. Some ARGs, including bla _CTX-M_ and *mcr-1*, are resistant to critical antibiotics and have often been found in mastitis bacterial isolates (Chehabi et al., 2019; Shafiq et al., 2021).

Driven by strong demand for dairy products, the dairy industry is growing worldwide (Grout et al., 2020). Intensive dairy management has been confirmed related to the increase in antibiotic consumption (Ferroni et al., 2020). Poor management of waste disposal was suggested to have influenced ARG prevalence on dairy farms (Dungan et al., 2018). However, ARGs and their bacterial hosts in dairy farm environments have not been well studied. In this study, we used metagenomic methodology to determine the presence of the ARGs in feces, wastewater, and soil samples collected from typical dairy farms in China; bioinformatics analysis was used to evaluate the similarity of ARG profiles and identify the potential ARG bacteria hosts in a variety of dairy farm environments.

## Materials and methods

### Sampling and pretreatment

In 2017 and 2018, a total of 21 dairy environment samples (seven each for feces, wastewater, and soil) were obtained from dairy farms across three provinces of North China: Beijing (BJ; *n* = 3), Tianjin (TJ; *n* = 2), and Hebei (HB; *n* = 2). On each farm, a total of 10 fresh feces collected from heifers, lactating cows, and dry-off cows were mixed as one sample to represent this farm. Wastewater was collected from sewage pools which converged the daily flushing water from the farm. Surface soil (0–15 cm) was collected from vegetable fields for which dairy manure was used for fertilization. The soil and tap water from the campus of the Chinese Academy of Agricultural Sciences (located downtown) as blank control (BC) samples. No blank feces control was set up because no cows on these farms were insulated from antibiotics during their own or their parent’s generation. All samples were transported to the laboratory on dry ice. In the lab, 50 ml of each wastewater sample was firstly cleaned by filter paper to remove the solid substance, then the filtrate was vacuum-filtered through a 0.22 μm cellulose ester membrane to intercept microorganisms, 2 l of lab tap water was directly through the 0.22 μm filter. The feces, soil, and filter membranes of water were kept at −80°C until further processing. The farms and sample information is summarized in Supplementary Table S1.

### DNA extraction and metagenomic sequencing

The genomic DNA of environmental samples was extracted using the Qiagen QIAamp DNA Stool Mini Kit (Qiagen, Germany) following the manufacturer’s instructions. DNA purity and integrity were analyzed by gel electrophoresis. DNA concentration was accurately quantified by Qubit 2.0 Fluorometer (Life Technologies, United States). A metagenomic library with an insert size of 350 bp was constructed by Novogene (Tianjin, China). The DNA extracted from BC water was below the detection limit. By increasing the sample quantity, little additional DNA quantity was obtained, so an optimized micro-library construction protocol was used for this sample (Jiang et al., 2015). Sequencing was performed on an Illumina HiSeq 4000 with the 150 bp paired-end sequencing strategy (Zhao et al., 2018). Raw reads were trimmed using Readfq (V8)^1^ to remove the reads containing (i) low-quality sequences ≥ a length of 38 bp; (ii) unknown base ≥10 bp; and (iii) overlap sequence with adapter ≥15 bp. On average, the metagenomic data size of 10 Gb and 32,501,357 clean reads were obtained for each sample.

### Metagenome assembly, ARG-like ORFs identification, and host annotation

The metagenomics sequences of each sample were *de novo* assembled with the default *k*-mer size through the CLC Genomics Workbench (version 10.0.1, CLC Bio, Aarhus, Denmark). A total of 2,154,023 contigs were obtained with an average length of 1,635 bp. The detailed information is summarized in Supplementary Table S2. Open reading frames (ORFs) were predicted within assembled contigs using Prodigal (version 2.6.0) (Hyatt et al., 2010). Then the ARG-like ORFs were searched against the deepARG database (Arango-Argoty et al., 2018) using BLASTP under the cutoff of *E* value ≤10^–5^, an ARG-like ORF was annotated if met the standard of sequence similarity ≥70% and hit length ≥ 25 amino acids (Zeng et al., 2019). The coverage of these ORFs was calculated using CLC Genomics Workbench (version 10.0.1), through the method of mapping metagenomic reads to the contigs with a minimum length coverage ≥95 at 95% similarity (Ma et al., 2016). ARG coverage, indicating the abundance (times per Giga base, ×/Gb) of ARG-like ORFs, as defined by the following equation:


$$\mathrm{Coverage}=\overset{n}{\underset{1}{\sum }}\frac{N\times 150/L}{S}$$

*N* indicates the number of reads mapped to ARG-like ORFs, *L* is the sequence length of target ARG-like ORFs, *n* is the number of ARG-like ORFs, 150 is the length of the Illumina sequencing reads, and *S* is the sequencing data size (Ma et al., 2016).

*Tet*(X)-like sequence was further genotyped against a *tet*(X) pool (Umar et al., 2021; Xu et al., 2021). The predicted ARG-like ORFs were compared against the NCBI NR database through BLASTP with the cutoff of *E* value ≤10^–5^, and then parsed by MEGAN (MetaGenome Analyzer, version 6). The annotation of taxonomic genus classification was assigned with the criteria of voting score > 50% (Zeng et al., 2019).

### Statistical analysis

Shannon index values were used to assess alpha diversity and visualized using GraphPad Prism 7. Principal component analysis (PCA) was performed based on the coverage of ARG types, to reflect the beta diversity of ARGs. Principal co-ordinates analysis (PCoA) was performed based on the Bray–Curtis distance to assess the beta diversity of microbiomes. Permutational multivariate analysis (Adonis) was performed to detect the difference among samples. Statistical comparisons were performed using the Mann–Whitney test (Zeng et al., 2019). *P* < 0.05 was defined as significantly different. PCA, PCoA Venn, and Pie chart were performed with R software version 3.6.3. Heatmaps, bar, and box charts were generated using ImageGP (Chen et al., 2022). Hierarchical cluster analysis adopted the weighted pair group method using arithmetic averages (WPGMA). The correlation between ARGs and bacteria was calculated by the Spearman rank method with FDR adjustment of the *P*-value. The correlation coefficient (*ρ*) ≥ 0.8 and significance level *P* < 0.05 level was recognized as significantly correlated and used to finalize the network by using Gephi (v0.9.3) platform.

## Results

### ARG types

**Figure 1:**
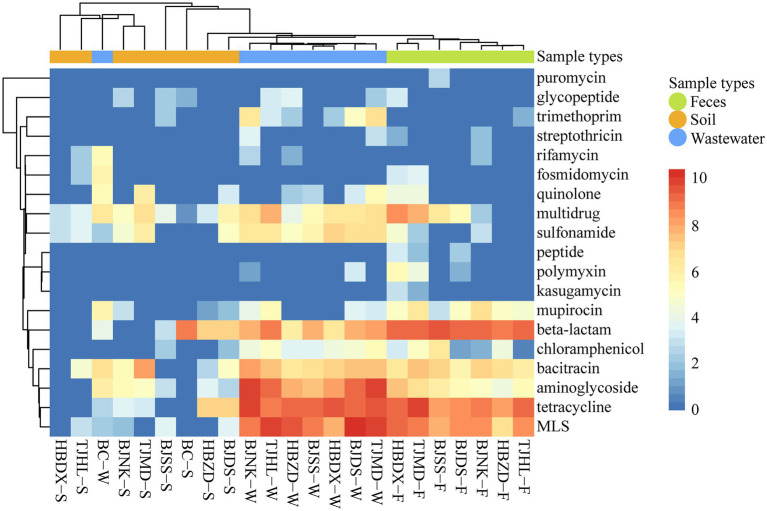
Heatmap of ARG types (coverage, × /Gb, log2 transferred) in 23 environmental samples. The combination of four letters (e.g., TJHL) represents a farm; BC, blank control; W, wastewater; F, feces; S, soil.

Among the 23 samples, a total of 19 types were annotated from the deepARG database (Figure 1). The abundance of ARG types varied across different environmental samples, from 9.63 × 10^–1^ copies/Gb (multidrug resistance genes in the BC soil) to 1.55 × 10^3^ copies/Gb (macrolide-lincosamide-streptogramin (MLS) resistance in wastewater). The genes resistant to tetracycline, MLS, and beta-lactam were the major resistance types and followed by aminoglycoside, bacitracin, and multidrug. Both BC soil and water had lower ARG richness than dairy farm environment samples.

**Figure 2:**
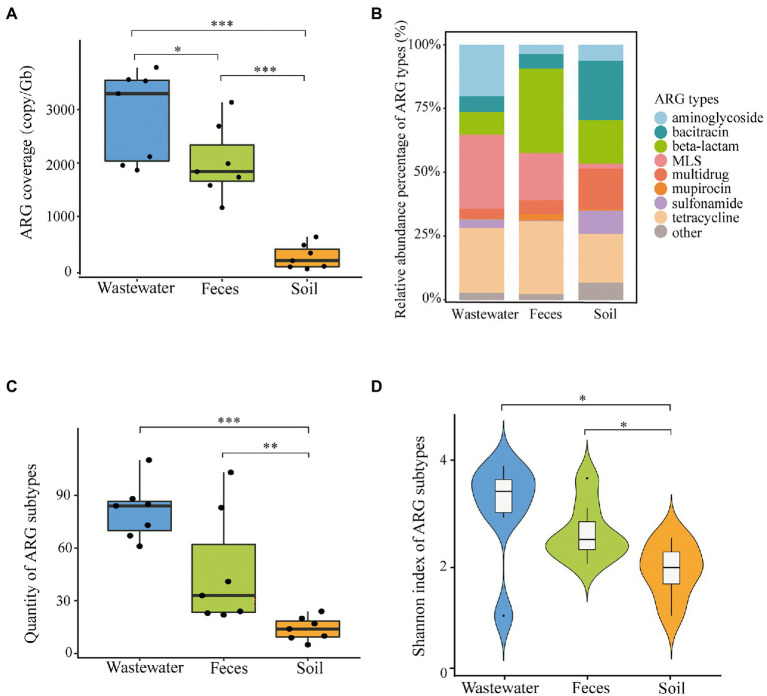
ARG profiles in dairy wastewater, feces, and soil. **(A)** Total ARGs coverage of each environmental sample. **(B)** ARG types composition of three sample types. **(C)** ARG subtypes quantity across three sample types. **(D)** Shannon index of ARG subtypes across three sample types. *P < 0.05; **P < 0.01; and ***P < 0.001.

The total ARG coverages of three kinds of dairy farm environment samples were significantly different (*P* < 0.05) (Figure 2A). Average ARG abundance of each wastewater sample (2.87 × 10^3^ ± 0.68 × 10^3^ copies/Gb) and feces (2.02 × 10^3^ ±0.85 × 10^3^ copies/Gb) were much higher than that of soil (0.24 × 10^3^ ± 0.23 × 10^3^ copies/Gb). The most dominant ARGs varied in feces (β-lactam 33.05%, tetracycline 28.51%, MLS 18.55%), wastewater (MLS 29.06%, tetracycline 25.43%, aminoglycoside 20.20%), and soil (bacitracin 23.22%, tetracycline 19.16%, β-lactam 17.05%) (Figure 2B). Multidrug and sulfonamide resistance genes occupied a higher proportion in soil (15.86% and 9.08%) than in feces and wastewater. The difference in ARGs distribution in dairy farm environments might be driven by the nature of selection under the different physicochemical properties of antibiotics and environmental conditions. It might also be impacted by anthropogenic activities such as the frequent use of certain antibiotics.

### ARG subtypes

**Figure 3:**
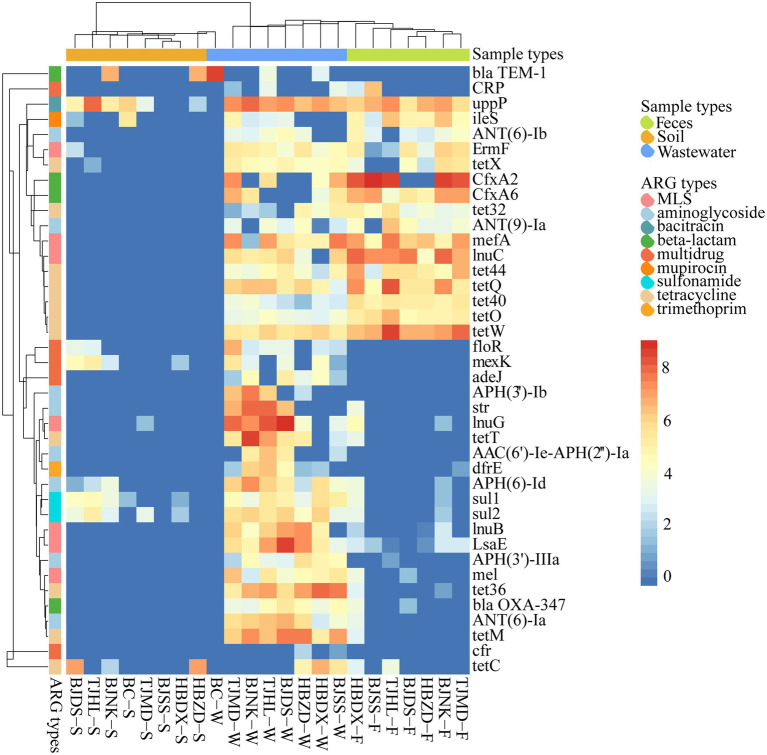
Heatmap of main ARG subtypes (coverage, × /Gb, log2 transferred) in 23 environmental samples. The combination of four letters (e.g., TJHL) represents a farm; BC, blank control; W, wastewater; F, feces; S, soil.

A total of 287 subtypes were detected in 21 dairy farm environment samples. The coverage of different subtypes ranged from 0.59 to 632.57 copies/Gb in feces, 0.68 to 569.47 copies/Gb in wastewater, and 0.81 to 277.86 copies/Gb in soil. The main ARG subtypes of different samples are shown in Figure 3. The quantity of ARG subtypes detected in wastewater and feces was comparable (*P* < 0.05) and significantly greater than in soil (Figure 2C). Wastewater had the most diverse and abundant ARG subtypes. On average, each sample of wastewater, fecal, and soil carried 80 ± 15, 46 ± 30, and 14 ± 6 kinds of ARG subtypes, respectively. There was no systemic difference between the Shannon index of feces and wastewater (*P* > 0.05), however, both of them were significantly higher than that of soil (*P* < 0.05) (Figure 2D).

The 10 representative ARG subtypes in feces, wastewater, and soil, which, respectively, belong to 5, 4, and 7 ARG types, are listed in Supplementary Table S3. These genes dominated the abundance among all subtypes and accounted for 72.6%, 40.52%, and 83.09% of total ARG coverage of feces, wastewater, and soil, respectively. The top 3 abundant subtypes varied in feces [*cfx*A2, *tet*(W), and *lnu*(C)], wastewater [*lnu*(G), *uppP*, and *tet*(M)] and soil [*uppP*, *tet*(C), and *bla*_TEM-1_]. We found that the dominant tetracycline resistance genes are completely different among these environments, such as feces with *tet*(W), *tet*(Q), *tet*(44), wastewater with *tet*(M), *tet*36, *tet*(T), and soil with *tet*(C). *Upp*P was the only shared ARG gene in the top 10 dominant ARGs of these three kinds of environment matrix.

*Tet*(X) was frequently detected in wastewater (7/7), feces (5/7), and soil (1/7) samples, with the coverage ranging from 20.53 to 47.42 copies/Gb in wastewater, and from 5.74 to 68.99 copies/Gb in feces, the soil had the lowest abundance at 2.72 copies/Gb. Five *tet*(X) genotypes were detected, including *tet*(X2), *tet*(X5), *tet*(X11), *tet*(X14), and *tet*(X18). *Cfr* was detected in wastewater samples (2/7) with 3.78 ± 1.64 copies/Gb, while not detected in feces and soil. Different van subtypes were founded in feces (1/7), wastewater (4/7), and soil (4/7) samples with a range of 0.90–8.70 copies/Gb.

### Comparison analysis of ARG profiles

**Figure 4:**
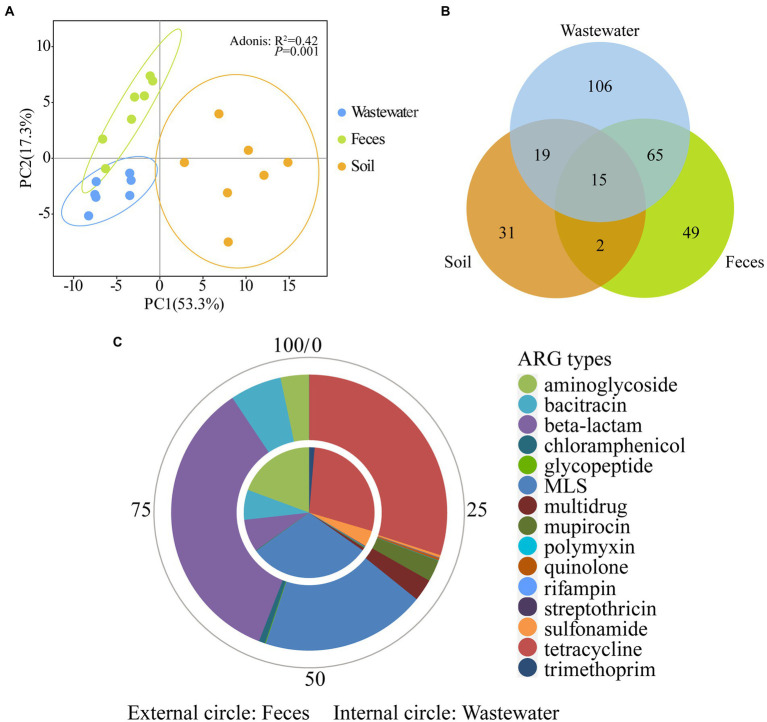
Comparison of ARGs across wastewater, feces, and soil in dairy farms. **(A)** PCA plots (coverage, × /Gb, log2 transferred) of 21 environmental samples. **(B)** Shared ARGs across three sample types. **(C)** The relative abundance of shared ARGs between wastewater and feces.

PCA analysis showed the 21 dairy farm samples clustered by environmental types (Adonis test, *R*^2^ = 0.42, *P* = 0.001) (Figure 4A), indicating the ARG profiles of feces, wastewater, and soil are different. Within each type, the feces and wastewater samples clustered closer, while soil samples scattered in a broad range, suggesting greater variation of ARG compositions across different soil samples. The feces group and wastewater group clustered closely and far from the soil group, implying a potentially higher correlation between wastewater and feces.

The numbers of shared and unique ARGs of feces, wastewater, and soil are shown in Figure 4B. A total of 80 ARGs belonging to 15 types were shared between feces and wastewater (Supplementary Table S4). These shared genes contributed to 96.30 ± 3.38% and 85.40 ± 5.20% of the total ARG coverage in feces and wastewater, respectively, further illustrating the similarity of ARG composition of wastewater and feces. Among these shared ARGs, genes resistant to MLS, tetracycline, and aminoglycoside were abundant in wastewater, whereas β-lactam, tetracycline, and MLS were abundant in feces (Figure 4C).

A total of 15 ARGs belonging to 7 types (Supplementary Table S5) were shared by feces, wastewater, and soil and contributed to 12.49 ± 4.39%, 18.40 ± 7.57%, and 51.76 ± 21.97% of the total ARG coverage, respectively (Supplementary Figure S1). Genes resistant to bacitracin were the most abundant shared ARGs and accounted for 35.21%, 46.91%, and 39.27% of the total shared ARG coverage in wastewater, feces, and soil samples, respectively. Besides bacitracin, among these shared genes, genes resistant to aminoglycoside and sulfonamide were abundant in wastewater, while mupirocin and MLS were abundant in feces, and tetracycline and sulfonamide were abundant in soil.

### Bacterial hosts of ARGs

The bacterial community composition showed that Firmicutes dominated bacterial phyla in dairy feces and wastewater (50.60 ± 3.51% and 29.07 ± 10.50%, respectively), followed by Bacteroidetes (20.33 ± 2.87% and 22.96 ± 7.30%, respectively) (Supplementary Figure S2A). Proteobacteria was the primary phylum in soil (45.03 ± 14.78%). At the genus level, *Bacteroides* dominated in feces and wastewater, while *Sphingomonas* dominated in soil (Supplementary Figure S2B). The PCoA analysis showed the microbiomes clustered by environmental types (Adonis test, *R*^2^ = 0.57, *P* = 0.001) (Supplementary Figure S3). All feces clustered closely implying a high similarity of gut microbiomes of cows among different farms.

A total of 10,204 ARG-like (ORFs) located in 8,344 assembled contigs were annotated at the genus level in all feces, wastewater, and soil samples (Supplementary Table S2). The major bacterial hosts in wastewater, feces, and soil and their harboring ARGs are summarized in Supplementary Table S6. In feces, the top 3 primary bacterial hosts including *Bacteroides*, *Bifidobacterium*, and *Escherichia*, totally harbored 73.5% of ARG coverage. In wastewater, *Bacteroides*, *Enterococcus*, and *Streptococcus* dominated and totally harbored 45.2% of ARG coverage. *Luteimonas*, *Enterococcus*, and *Escherichia* were the principal ARG hosts in soil and totally harbored 52.82% of ARG coverage. Notably, the major bacterial hosts Escherichia and Streptococcus were the common mastitis pathogens of dairy cows.

**Figure 5:**
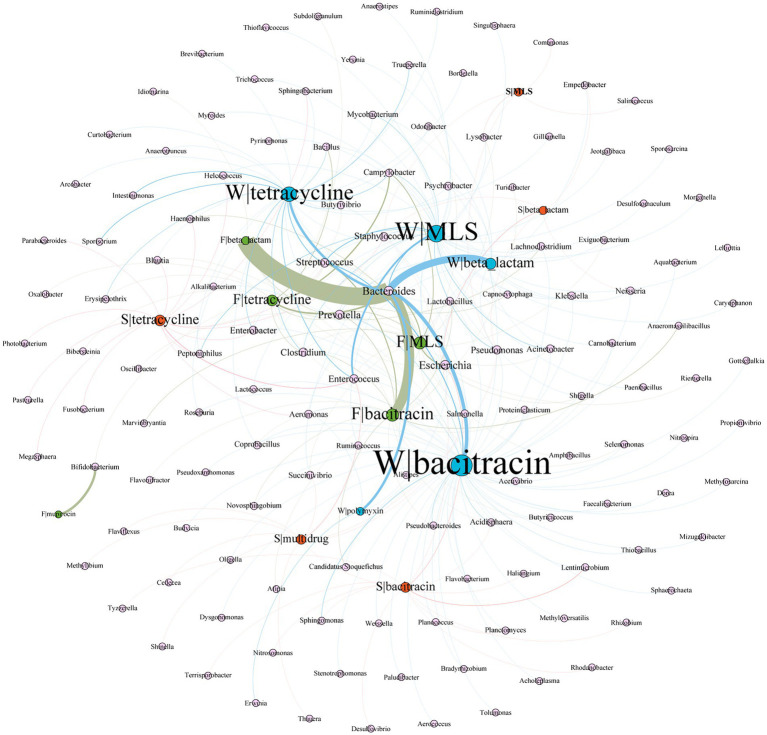
Network analysis of co-occurrence patterns between five major ARG types and major ARG hosts. The nodes were colored according to sample type (blue, wastewater; green, feces; orange, soil). The size of each node was proportional to the number of connections, i.e., the average weighted degree. W, wastewater; F, feces; S, soil. For example, F|tetracycline represents the resistance gene of tetracycline from feces.

Gene co-occurrence pattern analysis was performed on 5 major ARG types (tetracycline, MLS, β-lactam, bacitracin, and multidrug) against the bacterial hosts. As shown in Figure 5, *Bacteroides* play an important role in wastewater and feces. For example, *Bacteroides* harbored most of the diversity and abundance of β-lactam, bacitracin, tetracycline, and MLS resistant genes from wastewater and feces, especially, β-lactam resistance genes present the strongest correlation with *Bacteroides*. We also found that β-lactam, bacitracin, MLS, and tetracycline resistance genes have more bacterial hosts in wastewater than in feces. *Bifidobacterium* was the unique host of mupirocin resistance genes in feces.

## Discussion

Livestock and their environments have been evidently proved the large reservoirs for ARGs (Shi and Wang, 2018; Wang et al., 2019). Due to the rapid increase in the number of dairy cows, greater amounts of therapeutic or preventative antibiotics are being used in the dairy industry (Wemette et al., 2020). Consequently, dairy waste products such as feces and wastewater are increasing and inevitably emitted into environments such as soil. In this study, we used the metagenomic method to explore whether there were differences in the ARGs and bacterial hosts in dairy feces, wastewater, and soil, and whether there was a potential ARG dissemination trend on Chinese typical dairy farms where antibiotics were routinely used. Compared with conventional bacteria culture or the PCR method, the metagenomics method has the advantages of cultural independence and high throughput, and could provide a more comprehensive view of the resistomes of environments (Dos Santos et al., 2017).

Overall, the abundance and diversity of ARGs in dairy farm environmental samples were much higher than in control samples of tap water from the lab and soil from campus (Figure 1). This result is similar to a previous finding that conventional dairy farm environments presented significantly higher ARGs richness than organic dairy farms where no antibiotics were applied for at least 4 years(Pitta et al., 2020). It is referred that exposure to antibiotics might promote the emergency and persistence of ARG in dairy farm environments. ARGs of tetracyclines, MLS, β-lactams, and aminoglycosides, were found more abundant than other antibiotics such as quinolones and rifamycin in these dairy farm environments. This is consistent with the frequent antibiotic types used in these dairy farms. A similar association has also been reported between the class of dominant ARGs in dairy feces and the antibiotic class used (Rovira et al., 2019; Pitta et al., 2020). These data indicate the types and amount of antibiotics administrated to dairy cows might play an important role in shaping the antibiotic resistomes of the dairy farm environments.

Despite the dairy wastewater resistome having a greater ARG subtype number and abundance than feces (Figures 2A,C), the Shannon index showed their ARG profiles are comparable (*P* > 0.05) (Figure 2D). More shared genes between wastewater and feces were found comparing with Rovira’s findings (80 vs. 62) (Rovira et al., 2019), and these genes accounted for a very high proportion of abundance (80.20–99.68%) among all the wastewater and fecal samples. These outcomes imply feces might be the most important ARG source of wastewater. It is assumed that besides animal feces, other environments such as troughs and floors, might contain substantial numbers of ARGs and were flushed into the wastewater ponds. However, contrary to our finding that more ARG subtype numbers in wastewater than in feces (80 vs. 46), Rovira reported remarkably more ARG subtype numbers in dairy feces than in wastewater (118 vs. 47) (Rovira et al., 2019). This difference might be caused by various sampling time and effluent accumulation duration in the wastewater ponds. Because the persistence of ARGs in environments is liable to be impacted by natural conditions, e.g., the removal efficiency of ARGs in swine wastewater was higher in winter than in summer (Sui et al., 2017). Consistent with several reports (Rovira et al., 2019; Pitta et al., 2020), significantly fewer ARGs were detected in soil than in feces and wastewater on dairy farms. Pre-treatment of wastewater and feces, and the time registration before application to the soil might explain the fewer ARGs in the soil, these two methods were proved effective for the decrease of ARGs (Wind et al., 2021).

Tetracycline resistance genes were widely prevalent among dairy feces, wastewater, and soil (Figure 2B). This universality is in agreement with many other investigated cattle, swine, and poultry farm environments (Wang et al., 2018; Duan et al., 2019), and was suggested to correlate with the massive application of tetracycline. It has been reported that the annual consumption of tetracycline is 12,000 tons in China (Zhang et al., 2015). In dairy feces, 5 subtypes including *tet*(32), *tet*(40), *tet*(44), *tet*(O), *tet*(Q), and *tet*(W) were detected on each farm. Similar resistome prevalence trends were reported in gut microbiomes of other dairy and swine with *tet*(32), *tet*(44), *tet*(O), *tet*(Q), and *tet*(W), and in humans with *tet*(32), *tet*(O), *tet*(Q), and *tet*(W) (Forslund et al., 2013; Lim et al., 2020). Besides the same dominant subtypes *tet*(44), *tet*(M), *tet*(W), *tet*(Q), and *tet*(O) in swine feedlot wastewater (Wang et al., 2018), another 3 subtypes *tet*(36), *tet*(40), and *tet*(X) were also 100% prevalent in dairy wastewater. Few tetracycline-resistant subtypes including efflux pump genes *tet*(C) and *tet*(G) and the enzymatic modification gene *tet*(X) were detected in soil. Previous studies showed after manure anaerobic fermentation, the common ribosomal protection protein genes such as *tet*(O), *tet*(Q), *tet*(W), and *tet*(M) decreased significantly, while *tet*(G) had the least variation and *tet*(C) and *tet*(X) increased (Cheng et al., 2016; Qian et al., 2016).

Tigecycline is an alternative to colistin for treating serious multidrug-resistant antimicrobial infections. Though tigecycline has not been approved for animal use, the emergence of tigecycline resistance gene might be incubated by the extensive usage of tetracycline (Pan et al., 2020). In this study, a high positive rate of *tet*(X) was detected in the microbiomes of dairy environments, in agreement with the recent finding in cattle feces (Fu et al., 2021). The coverage of *tet*(X) in feces and wastewater was higher than in soil. *Tet*(X5) variant, which has been proven could mediate tigecycline resistance and spread in swine and poultry(Chen et al., 2021), was found in the present study and will be further validated in the following research. The primary hosts of detected *tet*(X) variants, including *Bacteroides*, *Escherichia*, and *Acinetobacter*, are also common human-associated microbiomes (Garcia-Garcera et al., 2017; Jung et al., 2017). These results imply the dairy farm environment is another potential area for tigecycline resistance genes and a risky dissemination source that could compromise the last antibiotic defense in humans.

In dairy feces, β-lactam was the most abundant resistance class (Figures 1, 2B). Rovira (Rovira et al., 2019) reported the similar top dominance of β-lactam resistance genes in dairy whereas not in beef feces. An investigation showed the consumption of β-lactam on dairy farms was around 10 times higher than on beef farms (Ferroni et al., 2020). The relatively lower abundance of β-lactam resistance gene in dairy wastewater and soil might be due to the instability of β-lactam in the environment (Rovira et al., 2019). Same with reported fecal resistomes of bovine, swine, and human (Gatica et al., 2019; Lim et al., 2020), the *cfx*A family, including *cfx*A2 and *cfx*A6, were found as the major contributing genes conferring β-lactam resistance in our study. Extended-spectrum β-lactamase (ESBLs) resistance genes, such as *bla*_OXA_, *bla*_TEM_, *bla*_SHV_, and *bla*_CTX-M_, can hydrolyze most β-lactams used in humans and animals (Lee et al., 2020). *bla*_OXA_ and *bla*_TEM_ were mainly found in dairy wastewater and soil with low abundance (Figure 3). These two kinds of genes were found only in conventional dairy farms but not in organic dairy farms (Rovira et al., 2019; Pitta et al., 2020). No *bla*_SHV_ and *bla*_CTX-M_ were detected in this study.

In dairy wastewater, MLS resistance genes took up the greatest proportion of total ARG abundance. Among them, inactivation genes contribute to 51% MLS coverage and are followed by efflux (20%). A similar distribution profile was shown in feces, while few MLS resistance genes were detected in soil. In contrast to a previous study, the MLS efflux pump was most abundant in dairy agroecosystems (Pitta et al., 2016). This indicated even in similar breeding environments, ARGs profiles could present totally different and might be affected by complex factors. A total of 37 MLS subtypes were detected, while *lnu*(G) and *lnu*(C) were the predominant subtypes in dairy wastewater and feces, respectively. In another research, the *lnu*(C) also be found as the most abundant MLS resistance gene in dairy environments (Pitta et al., 2020). Interestingly, *erm*(F), was the single ribosomal methylase gene in feces, and the sole shared MLS gene among feces, wastewater, and soil. *Erm*(F) was mainly hosted in *Bacteroides*, and consistent with another study, in which *erm*(F) was the dominant gene among all erm genes located in *Bacteroides* (Johnsen et al., 2017).

Though significantly less ARGs than feces and wastewater, dairy soil still presented more abundant and diverse ARGs than BC soil (Figure 3). Similar results were found in the soil amended by dairy manure or wastewater (Dungan et al., 2018; Chen et al., 2019). *UppP* (renamed from *bacA*), a bacitracin-resistant gene, was widely distributed among different dairy environments and dominated the ARG abundance in dairy soil (Figure 3). As an undecaprenyl pyrophosphate phosphatase, *uppP* is a conserved protein in bacteria, can be found in numerous bacterial genera (Matos et al., 2009; Feng et al., 2018; Jukic et al., 2022). In our study, a total of 62 different genera were annotated as *upp*P bacterial hosts, confirming it is ubiquitous in multiple bacteria. Multidrug-resistant genes also contributed a higher proportion of ARG abundance in dairy soil (Figure 2B). A total of 74 subtypes of multidrug resistance genes were found among all samples, and these genes account for 7.95%, 3.69%, and 15.62% of total ARG coverage in feces, wastewater, and soil, respectively. *Cfr*, an rRNA methyltransferase, could mediate the combined resistance to phenicols, lincosamides, oxazolidinones, pleuromutilins, and streptogramin A (Long et al., 2006; Kaminska et al., 2010), was only found in dairy wastewater. The prevalence of *cfr* was less reported in dairy farms (Liu et al., 2019), but was often detected in swine farms(Wang et al., 2015; Huang et al., 2019). Although *mcr-1* was not detected in these dairy farm environments, the coexistence of *cfr* and *mcr-1* occurred in the same plasmid among the *E. coli* isolates from swine farms, suggesting that multidrug-resistant bacteria in dairy farm environments may acquire the *mcr-1 via* HGT. Therefore, the risk of colistin resistance spread is mounting (Ma et al., 2021).

Our findings revealed both resistomes and microbiomes are similar among the same environment type, regardless that these samples were collected from different dairy farms at different locations. We suspected the cow gut microbiomes might largely contribute to the similarity of ARGs in feces, and these resistomes were further transmitted to wastewater. For example, Firmicutes and Bacteroidetes were widely reported as the predominant phyla in cattle feces, and both account for approximately 70% of microbiomes (Zaheer et al., 2019), consistent with the present results. The genus of *Bacteroides* was identified as the dominant ARG host in dairy feces and wastewater. The ARG compositions carried by *Bacteroides* in dairy feces and wastewater were similar. Another study also reported *Bacteroides* carried similar ARGs in different dairy ecosystem samples (Pitta et al., 2016). β-lactam resistance genes being most abundant and followed by tetracycline and MLS among all *Bacteroides* carried ARGs. A previous study showed *Bacteroides* increase was associated with the increase of β-lactam resistance in the feces of ceftiofur-treated cows (Chambers et al., 2015). This evidence suggested that the bacterial host is a crucial factor in ARG profile shaping and their horizontal transfer in different environments.

We also found that the same ARGs were harbored by bacteria with different preferences in different environments. For example, the multidrug ARGs were mainly harbored by *Acinetobacter* and *Pseudomonas* (33% and 30%) in wastewater, by *Bifidobacterium* and *Escherichia* (50% and 40%) in feces, and by *Lysobacter* and *Pseudomonas* (48% and 36%) in soil. *Streptococcus* predominantly harbored MLS and tetracycline resistance genes in both feces (81% and 18%) and wastewater (55 and 43%). And, 97% β-lactam, 58% aminoglycoside, 57% bacitracin, 34% tetracycline resistance genes in feces, and 83% β-lactam and 61% bacitracin resistance genes in wastewater were harbored by *Bacteroides*. Monitoring antibiotic-resistant bacteria would be useful to help veterinarians prescribe effective medications and provide a basis for the control of ARG dissemination.

## Conclusion

Based on the metagenomics method, a broad ARG distribution and microbiome diversity were found in typical Chinese dairy feces, wastewater, and soil. The dominant ARG profiles among these dairy environments correspond to the commonly used antibiotic types in dairy cows. Dairy wastewater had the richest ARGs among these environments, of which feces might be an important ARG source. The ARGs in dairy soil were lower but still higher than in BC soil. Applying dairy feces and wastewater in farmland impacted the ARG profiles in soil. A high detection rate of *tet*(X) and the finding of *tet*(X5) suggested the potential challenges of spreading tigecycline resistance from dairy sources to humans. The same environmental sample type from different farms in different locations presented similar profiles of resistome and microbiome. Microbiota characteristics of dairy cows themselves and the ARG bacterial hosts might contribute to this phenomenon. Further investigation on the temporal and spatial change of ARGs and bacteria on more dairy farms should be conducted. *Bacteroides* was the most important ARG host in the present study, which might facilitate the HGT of ARGs in different environments. These results suggest the rational use of antibiotics in dairy cows, pretreatment of dairy wastes before application in farmland, and strengthening the research and surveillance on ARG bacterial hosts, would be helpful for the control of ARG dissemination in typical Chinese dairy farm environments.

## Data availability statement

Metagenomic sequencing data that support the findings of this study have been deposited in the NCBI Sequence Read Archive under accession number PRJNA860353.

## Author contributions

WX and XL contributed to the initial study conception and supervised the study. JK and YL completed the experimental implementation. XC, FX, and HW provided support in the sample and data collection. JK, YL, WX, and XL analyzed and interpreted all results. JK wrote the final manuscript. All authors contributed to the article and approved the submitted version.

## Funding

This work was supported by National Key Research and Development Program (2021YFD1800700) and Innovation Project of Chinese Academy of Agricultural Sciences (CAAS-FRI-06).

## Conflict of interest

The authors declare that the research was conducted in the absence of any commercial or financial relationships that could be construed as a potential conflict of interest.

## Publisher’s note

All claims expressed in this article are solely those of the authors and do not necessarily represent those of their affiliated organizations, or those of the publisher, the editors and the reviewers. Any product that may be evaluated in this article, or claim that may be made by its manufacturer, is not guaranteed or endorsed by the publisher.

## Supplementary material

The supplementary material for this article can be found online at: https://www.frontiersin.org/articles/10.3389/fmicb.2022.990272/full#supplementary-material

## Footnotes

^1^ https://github.com/cjfields/readfq
