# Supplementary figures and images for: Metagenomic insights into the antibiotic resistomes of typical Chinese dairy farm environments
Source: Front Microbiol. 2022 Sep 28;13:990272. doi: 10.3389/fmicb.2022.990272 (PMC9555277; doi:10.3389/fmicb.2022.990272)

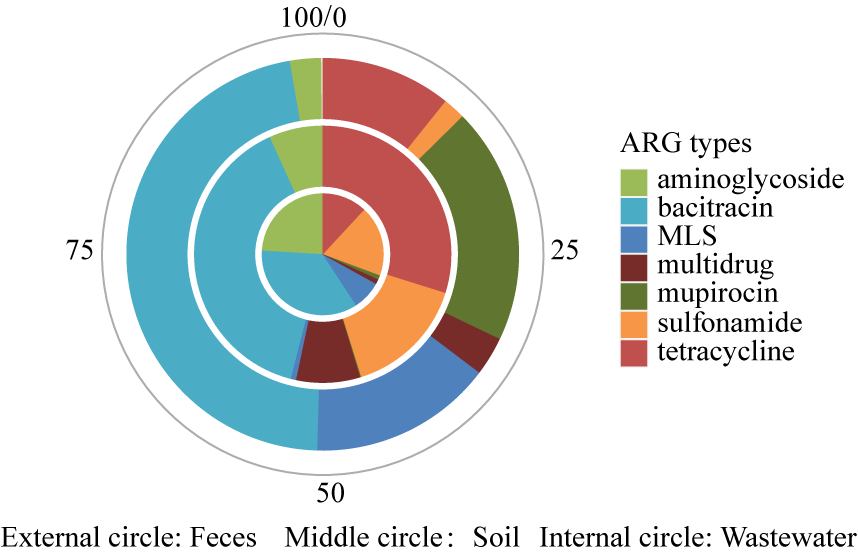

Supplement: Supplementary file 2 [file Image_1.TIF]

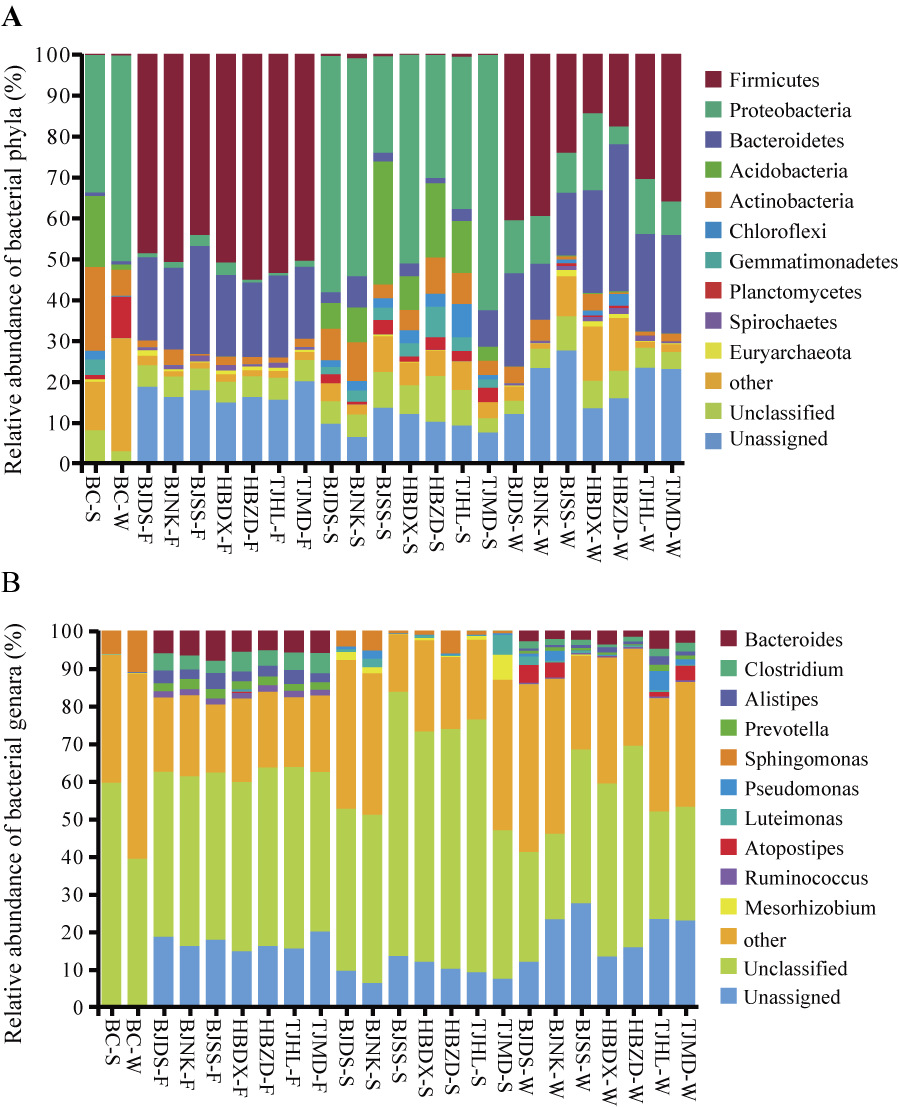

Supplement: Supplementary file 3 [file Image_2.TIF]

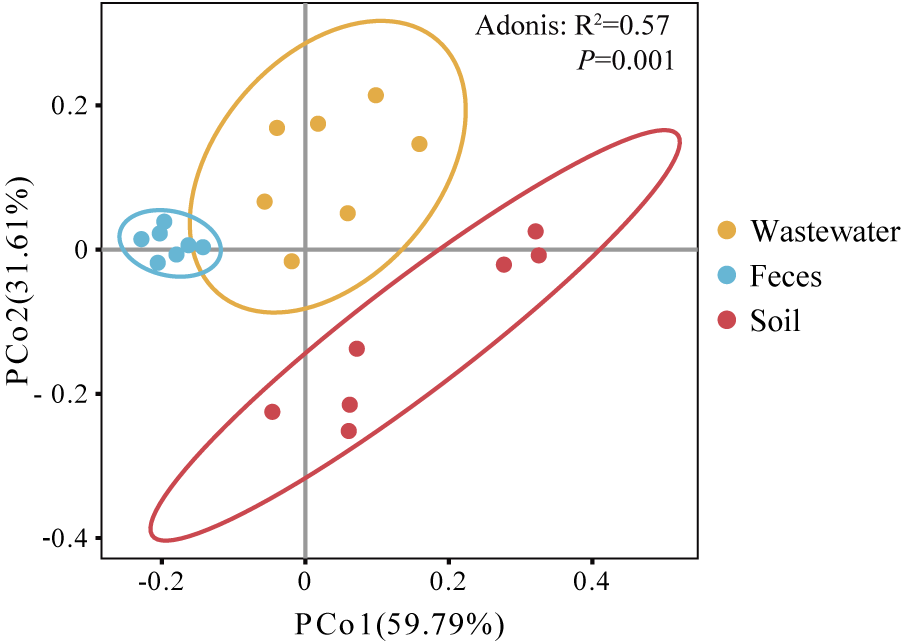

Supplement: Supplementary file 4 [file Image_3.TIF]
